# Mesenchymal stromal cells attenuate alveolar type 2 cells senescence through regulating NAMPT-mediated NAD metabolism

**DOI:** 10.1186/s13287-021-02688-w

**Published:** 2022-01-10

**Authors:** Xiaofan Lai, Shaojie Huang, Sijia Lin, Lvya Pu, Yaqing Wang, Yingying Lin, Wenqi Huang, Zhongxing Wang

**Affiliations:** 1 Department of Anesthesiology, The First Affiliated Hospital, Sun Yat-sen University, Guangzhou, China; 2 Zhongshan School of Medicine, Sun Yat-sen University, Guangzhou, China

**Keywords:** Pulmonary fibrosis, Mesenchymal stromal cells, Alveolar type 2 cells, Senescence, NAMPT

## Abstract

**Background:**

Idiopathic pulmonary fibrosis (IPF) is a chronic and progressive deadly fibrotic lung disease with high prevalence and mortality worldwide. The therapeutic potential of mesenchymal stem cells (MSCs) in pulmonary fibrosis may be attributed to the strong paracrine, anti-inflammatory, anti-apoptosis and immunoregulatory effects. However, the mechanisms underlying the therapeutic effects of MSCs in IPF, especially in terms of alveolar type 2 (AT2) cells senescence, are not well understood. The purpose of this study was to evaluate the role of MSCs in NAD metabolism and senescence of AT2 cells in vitro and in vivo.

**Methods:**

MSCs were isolated from human bone marrow. The protective effects of MSCs injection in pulmonary fibrosis were assessed via bleomycin mouse models. The senescence of AT2 cells co-cultured with MSCs was evaluated by SA-β-galactosidase assay, immunofluorescence staining and Western blotting. NAD+ level and NAMPT expression in AT2 cells affected by MSCs were determined in vitro and in vivo. FK866 and NAMPT shRNA vectors were used to determine the role of NAMPT in MSCs inhibiting AT2 cells senescence.

**Results:**

We proved that MSCs attenuate bleomycin-induced pulmonary fibrosis in mice. Senescence of AT2 cells was alleviated in MSCs-treated pulmonary fibrosis mice and when co-cultured with MSCs in vitro. Mechanistic studies showed that NAD+ and NAMPT levels were rescued in AT2 cells co-cultured with MSCs and MSCs could suppress AT2 cells senescence mainly via suppressing lysosome-mediated NAMPT degradation.

**Conclusions:**

MSCs attenuate AT2 cells senescence by upregulating NAMPT expression and NAD+ levels, thus exerting protective effects in pulmonary fibrosis.

**Supplementary Information:**

The online version contains supplementary material available at 10.1186/s13287-021-02688-w.

## Background

Idiopathic pulmonary fibrosis (IPF) is a chronic progressive respiratory disease with high morbidity and mortality [1, 2]. It is mainly characterized by epithelial cells injury, fibroblasts activation and continuing collagen accumulation in lung tissues [3, 4]. Until now there are no effective and approved medical treatments for IPF partly due to the unclear molecular mechanisms [5]. Thus, it is of great urgency to investigate the underlying mechanisms and develop better therapeutic strategies for IPF.

A growing body of evidence implicates the significance of cellular senescence during the pathogenesis of IPF [6]. Common senescence biomarkers including p16, p21 and senescence-associated β-galactosidase activity (SA-β-gal) have been detected in alveolar epithelial cells from IPF lung tissues [7, 8]. Senescent epithelial cells could secrete high levels of growth factors, cytokines and chemokines, which promote abnormal myofibroblasts differentiation and persistent tissue remodeling [9]. Fibrogenic exposures to irradiation could lead to alveolar type 2 (AT2) cells senescence and pulmonary fibrosis by inducing NOX-dependent oxidative stress [10]. Recently, Barry R. Stripp’s group reported that AT2 cells isolated from IPF lungs exhibit apparent features of cellular senescence, and senescence of AT2 cells facilitates progressive pulmonary fibrosis [11]. Targeting AT2 cells senescence might be an attractive strategy for preventing and attenuating pulmonary fibrosis.

Mesenchymal stromal cells (MSCs) have emerged as one of the leading candidates in cell-based therapeutic strategy for organ fibrosis due to their self-renewal capacity, multipotency and contribution to epithelial tissue repair [12]. KOWIT-YU CHONG et al. found that oncostatin M-preconditioned MSCs improved pulmonary function, reduced inflammatory and fibrotic mediators in bleomycin-Induced pulmonary fibrosis models through hepatocyte growth factor [13]. Transplantation of adipose-derived mesenchymal stem cells attenuates silicosis-induced pulmonary fibrosis via its anti-inflammatory and anti-apoptosis effects [14]. In addition, MSCs reduce endoplasmic reticulum stress mainly by PERK-Nrf2 pathway in a bleomycin-induced pulmonary fibrosis [15]. Moreover, human adipose-derived MSCs can inhibit the induction of EMT via Wnt/β-catenin pathway and repair radiation-induced pulmonary fibrosis [16]. However, there is still a lack of comprehensive understanding of the mechanisms underlying the therapeutic effects of MSCs in IPF, especially in terms of AT2 cells senescence.

Here, we investigated how MSCs therapy can improve pulmonary fibrosis in mouse models, specifically via modulating AT2 cells senescence. We demonstrated the potential role of MSCs in regulating the NAMPT-mediated NAD levels and senescence of AT2 cells.

## Results

### MSCs attenuate bleomycin-induced pulmonary fibrosis in mice

**Fig. 1:**
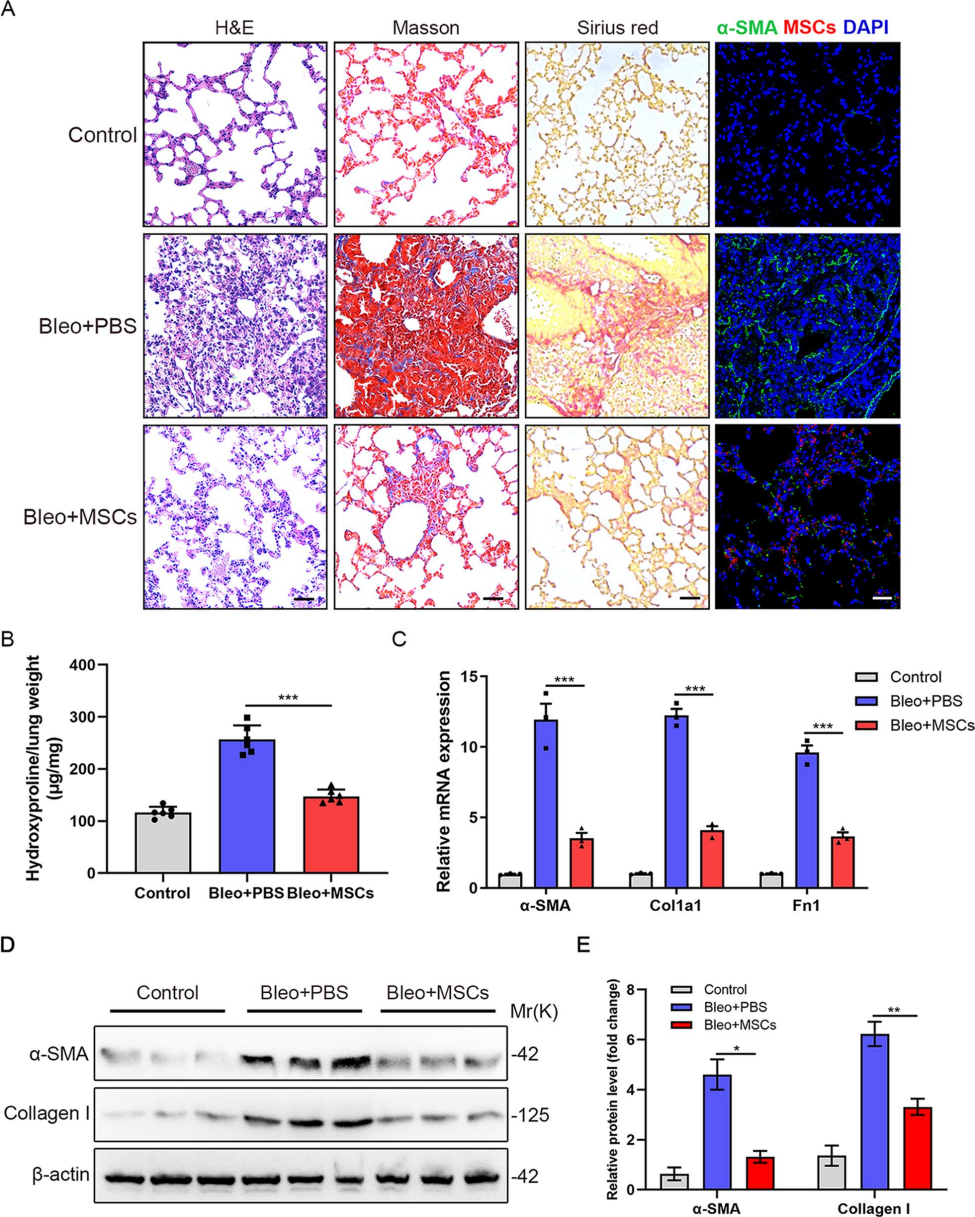
MSCs attenuate bleomycin-induced pulmonary fibrosis in mice. **A** H&E staining, Masson’s trichrome staining, Sirius red staining and representative immunofluorescence images obtained using an anti-α-SMA (green) antibody in lung sections from PBS-treated, Bleomycin-treated and Bleomycin + tdTomato^+^MSCs-treated mice (*n* = 6 per group). Scale bars: 50 µm. **B** Hydroxyproline levels in lungs of C57/BL6 mice from the different groups (*n* = 6 mice per group). **C** qPCR analysis of α-SMA, Col1a1 and Fn1 mRNA expression in lung slices from the different groups. **D** Western blot analysis of α-SMA and Collagen I in lungs from the different groups (*n* = 3 mice per group). **E** Quantification of α-SMA and Collagen I from D. Data are presented as the mean ± SEM; *P < 0.05, **P < 0.01, ***P < 0.001; one-way ANOVA and Tukey’s multiple comparisons test

Firstly, we used the murine bleomycin-induced pulmonary fibrosis model to assess the therapeutic capacity of MSCs. The administration of MSCs markedly attenuated bleomycin-induced pulmonary fibrosis, which was evidenced by H&E, Masson’s trichrome and Sirius red staining as well as hydroxyproline assays (Fig. 1A, B and Additional file 1: Figure S2A). We found that bleomycin-treated mice showed significant weight loss, while weight loss in MSCs-treated mice was significantly reversed relatively (Additional file 1: Figure S2B). Besides, compared to the Bleo-group, the survival and food intake of the MSCs group was significantly improved (Additional file 1: Figure S2C, D). Immunofluorescence staining also revealed that MSCs injection dramatically downregulated the bleomycin-induced expression of α-SMA in lung tissues (Fig. 1A). Moreover, α-SMA, Collagen I and fibronectin expression in lung tissues from the Bleo + MSCs group were significantly lower than those from the Bleo + PBS group (Fig. 1C–E). In summary, MSCs could attenuate bleomycin-induced pulmonary fibrosis in mice.

### Senescence markers are downregulated in AT2 cells from MSCs-treated pulmonary fibrosis mice

**Fig. 2:**
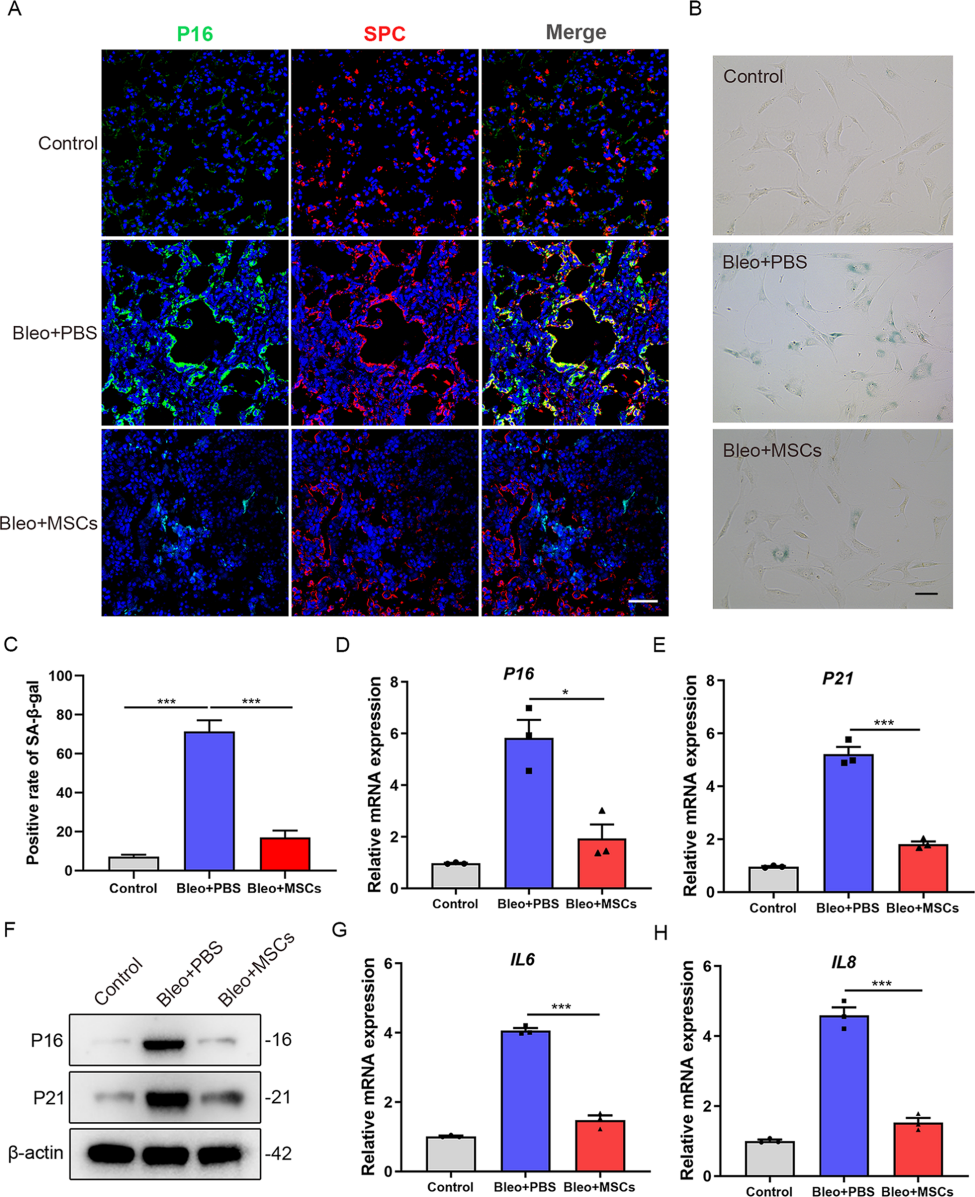
Senescence markers are downregulated in AT2 cells of MSCs-treated pulmonary fibrosis mice. **A** Immunofluorescence staining of lung sections from mice (*n* = 6 per group) and visualized using anti-P16 (green) and anti-SPC (red) antibodies. Scale bars: 50 µm. **B** SA-β-galactosidase staining of primary AT2 cells from mice of the different groups (*n* = 6 mice per group). **C** Quantification of the percentage of β-galactosidase positive cells from B. **D** qPCR analysis of P16 mRNA expression in primary AT2 cells from mice of the different groups. **E** qPCR analysis of P21 mRNA expression in primary AT2 cells from mice of the different groups. **F** Western blot analysis of P16 and P21 expression in primary AT2 cells from mice of the different groups. **G** qPCR analysis of IL6 mRNA expression in primary AT2 cells from mice of the different groups. **H** qPCR analysis of IL8 mRNA expression in primary AT2 cells from mice of the different groups. Data are presented as the mean ± SEM of three independent experiments; *P < 0.05, ***P < 0.001; one-way ANOVA and Tukey’s multiple comparisons test

It was reported that senescence of AT2 cells plays a crucial role in the development of progressive pulmonary fibrosis [11, 17]. Interestingly, we observed that treatment with MSCs significantly reduced the expression of the senescence marker p16 in AT2 cells (Fig. 2A). To further investigate whether MSCs exert an inhibiting effect on AT2 cells senescence, we isolated primary AT2 cells from mouse lungs of all groups. SA-β-galactosidase staining showed that there was an increase of SA-β-galactosidase positive rate in fibrotic AT2 cells compared with healthy cells, and SA-β-galactosidase positive rate was relatively decreased in MSCs-treated AT2 cells (*P* < 0.001) (Fig. 2B, C). Besides, freshly isolated primary AT2 cells from MSCs-treated lungs showed decreased P16 (*P* < 0.05) and P21 (*P* < 0.001) transcript levels relative to Bleo + PBS group (Fig. 2D, E). This was further confirmed by Western blot analysis (Fig. 2F). Besides, we further analyzed interleukin-6 (IL-6) and interleukin-8 (IL-8) levels, which are both major components of the senescence-associated secretory phenotype (SASP), and found both were significantly decreased upon MSCs treatment (Fig. 2G, H). Together, the above data suggested that MSCs treatment reduced the senescence of AT2 cells from mice lungs.

### AT2 cells co-cultured with MSCs decrease senescence markers

**Fig. 3:**
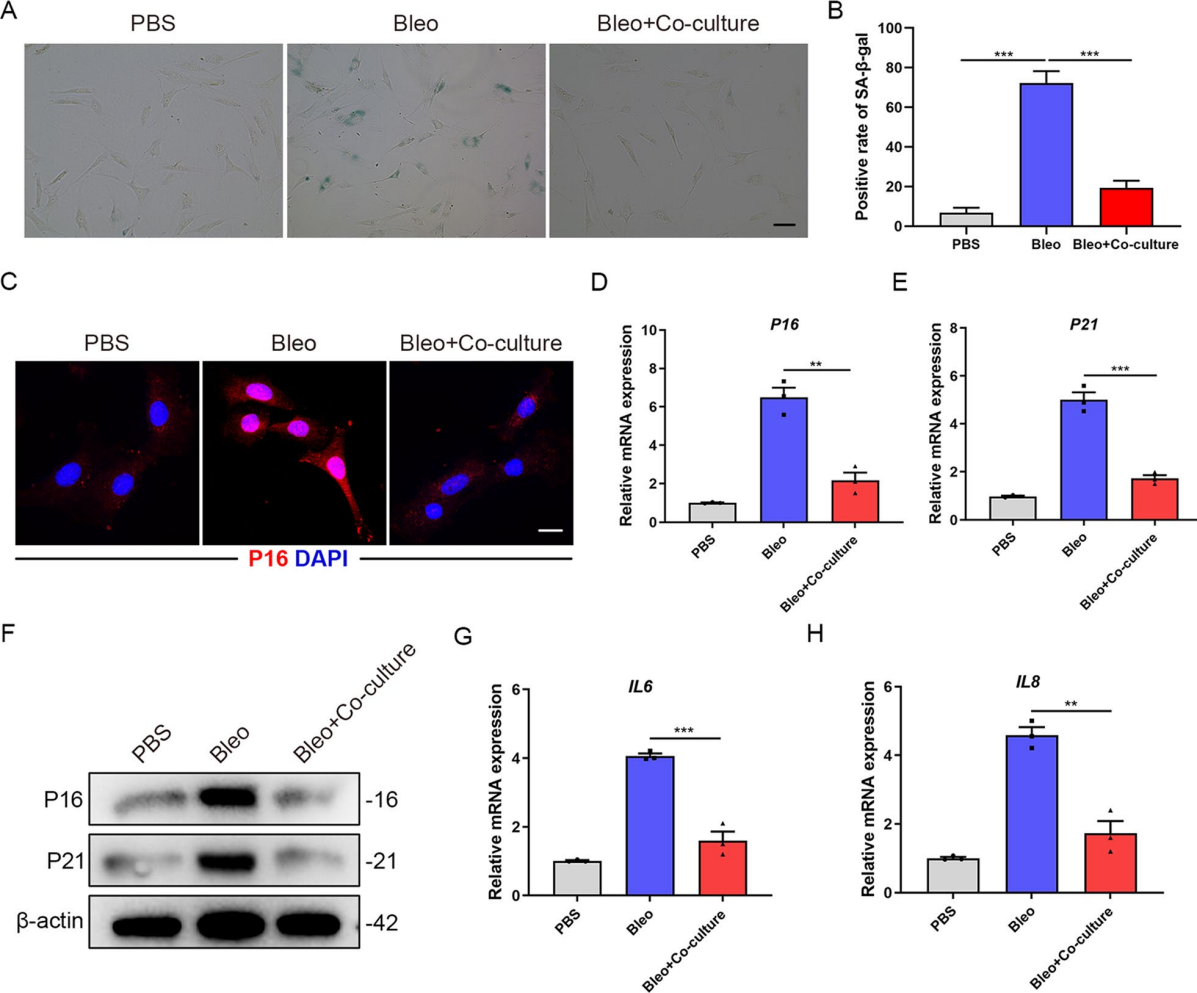
AT2 cells co-cultured with MSCs decreases senescent markers. **A** SA-β-galactosidase staining of primary AT2 cells co-cultured with MSCs. **B** Quantification of the percentage of β-galactosidase positive cells from A. **C** Immunofluorescence staining of primary AT2 cells co-cultured with MSCs and visualized using anti-P16 (red) antibody. Scale bars: 10 µm. **D** qPCR analysis of P16 mRNA expression in primary AT2 cells co-cultured with MSCs. **E** qPCR analysis of P21 mRNA expression in primary AT2 cells co-cultured with MSCs. **F** Western blot analysis of P16 and P21 expression in primary AT2 cells co-cultured with MSCs. **G** qPCR analysis of IL6 mRNA expression in primary AT2 cells co-cultured with MSCs. **H** qPCR analysis of IL8 mRNA expression in primary AT2 cells co-cultured with MSCs. Data are presented as the mean ± SEM of three independent experiments; **P < 0.01, ***P < 0.001; one-way ANOVA and Tukey’s multiple comparisons test

To further confirm that MSCs play a key role in regulating AT2 cells senescence, we isolated primary AT2 cells from wild-type mice and adopted the co-culture system as previously described [18]. We found a reduction of SA-β-galactosidase positive rate in AT2 cells co-cultured with MSCs compared with bleomycin-treated cells (*P* < 0.001) (Fig. 3A, B). We observed that bleomycin-induced P16 upregulation was inhibited in AT2 cells co-cultured with MSCs via immunofluorescence staining (Fig. 3C). In addition, qPCR and Western blot analyses revealed that there were decreased P16 (*P* < 0.01) and P21 levels (*P* < 0.001) in AT2 cells co-cultured with MSCs (Fig. 3D–F). Additionally, IL-6 and IL-8 levels were significantly decreased upon MSCs co-culture (Fig. 3G, H). Similar results were found in A549 cells (Additional file 1: Figure S3A-C). Together, the above data indicated that MSCs co-culture could suppress the senescence of AT2 cells in vitro.

### NAD+ and NAMPT levels are restored in AT2 cells co-cultured with MSCs

**Fig. 4:**
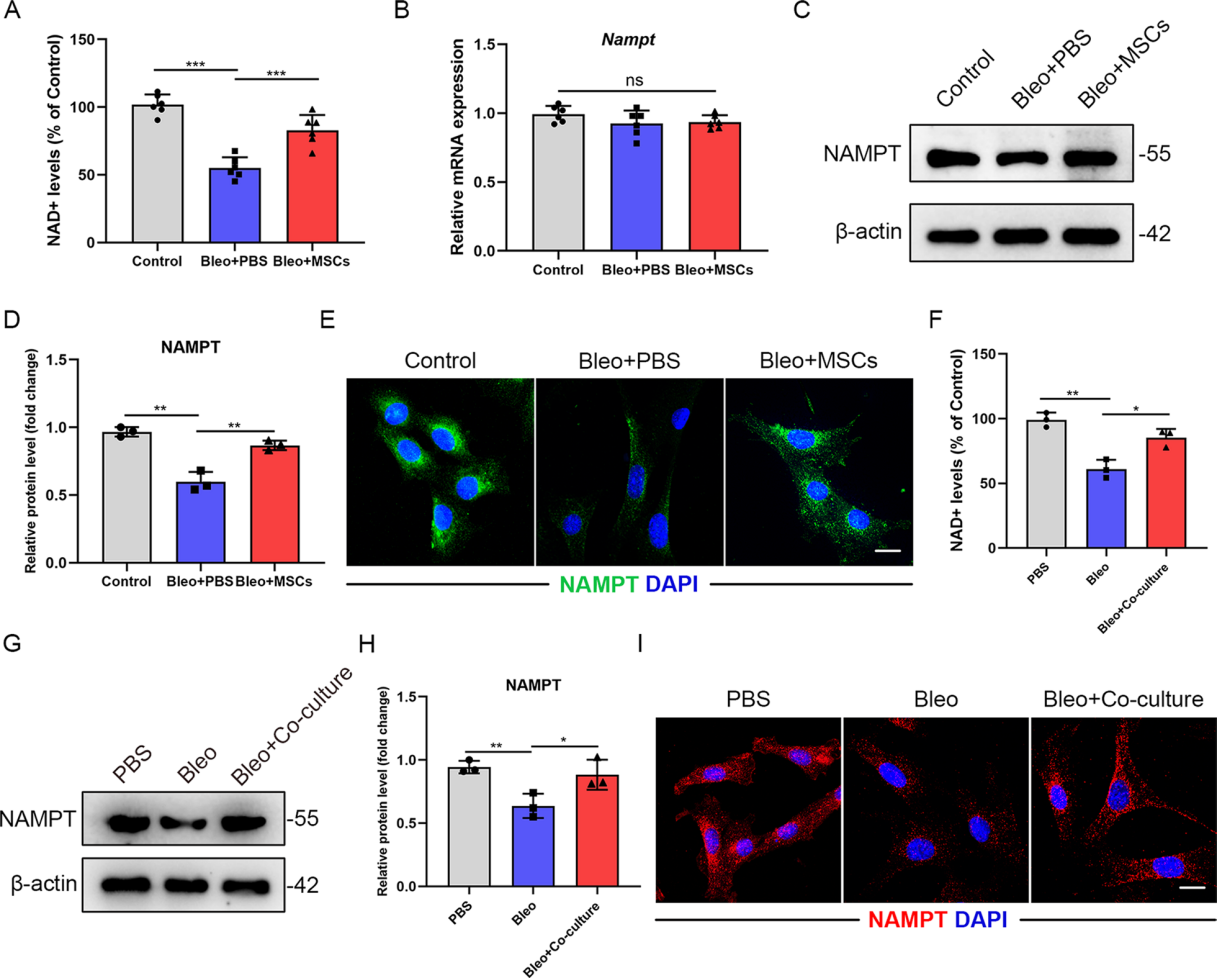
NAD+ and NAMPT levels are restored in AT2 cells co-cultured with MSCs. **A** NAD+ levels (% of Control) of primary AT2 cells from mice of the different groups (*n* = 6 mice per group). **B** qPCR analysis of Nampt mRNA expression in primary AT2 cells from mice of the different groups (*n* = 6 mice per group). **C** Western blot analysis and **D** quantification of NAMPT expression in primary AT2 cells from mice of the different groups. **E** Immunofluorescence staining of primary AT2 cells from mice of the different groups and visualized using anti-NAMPT (green) antibody (*n* = 6 mice per group). Scale bars: 10 µm. **F** NAD+ levels (% of Control) of primary AT2 cells co-cultured with MSCs. **G** Western blot analysis and **H** quantification of NAMPT expression in primary AT2 cells co-cultured with MSCs. **I** Immunofluorescence staining of primary AT2 cells from mice of the different groups and visualized using anti-NAMPT (red) antibody. Scale bars: 10 µm. Data are presented as the mean ± SEM of three independent experiments; *P < 0.05, **P < 0.01, ***P < 0.001; one-way ANOVA and Tukey’s multiple comparisons test

Abundant literature demonstrated that Nicotinamide adenine dinucleotide (NAD+) is a coenzyme found in all living cells and NAD+ levels decline during aging and senescence [19]. NAD+ can affect a number of important cellular processes, including cellular senescence, metabolic pathways and immune cell function [20]. We detected a significant reduction of NAD+ levels in fibrotic AT2 cells, while the NAD+ levels showed an increase in MSCs-treated AT2 cells relatively (*P* < 0.001) (Fig. 4A). NAMPT, the rate-limiting enzyme in the NAD+ salvage pathway, has been proven to be a key player in regulating NAD+ levels [21, 22]. Surprisingly, the protein levels of NAMPT decreased in AT2s from fibrotic lungs and it was apparently restored in AT2s from MSCs-treated fibrotic lungs (*P* < 0.01), but its mRNA levels remained unchanged among groups (Fig. 4B–D). This phenomenon was further validated by immunofluorescence staining (Fig. 4E). MSCs co-culture experiments also showed that there was an upregulation of NAMPT protein levels (*P* < 0.05) but not mRNA levels in AT2s co-cultured with MSCs (Fig. 4F–I and Additional file 1: Figure S4A). Together, MSCs co-culture could rescue the downregulation of NAD+ and NAMPT levels in senescent AT2 cells.

### MSCs inhibit AT2 cells senescence by suppressing lysosome-mediated NAMPT degradation

**Fig. 5:**
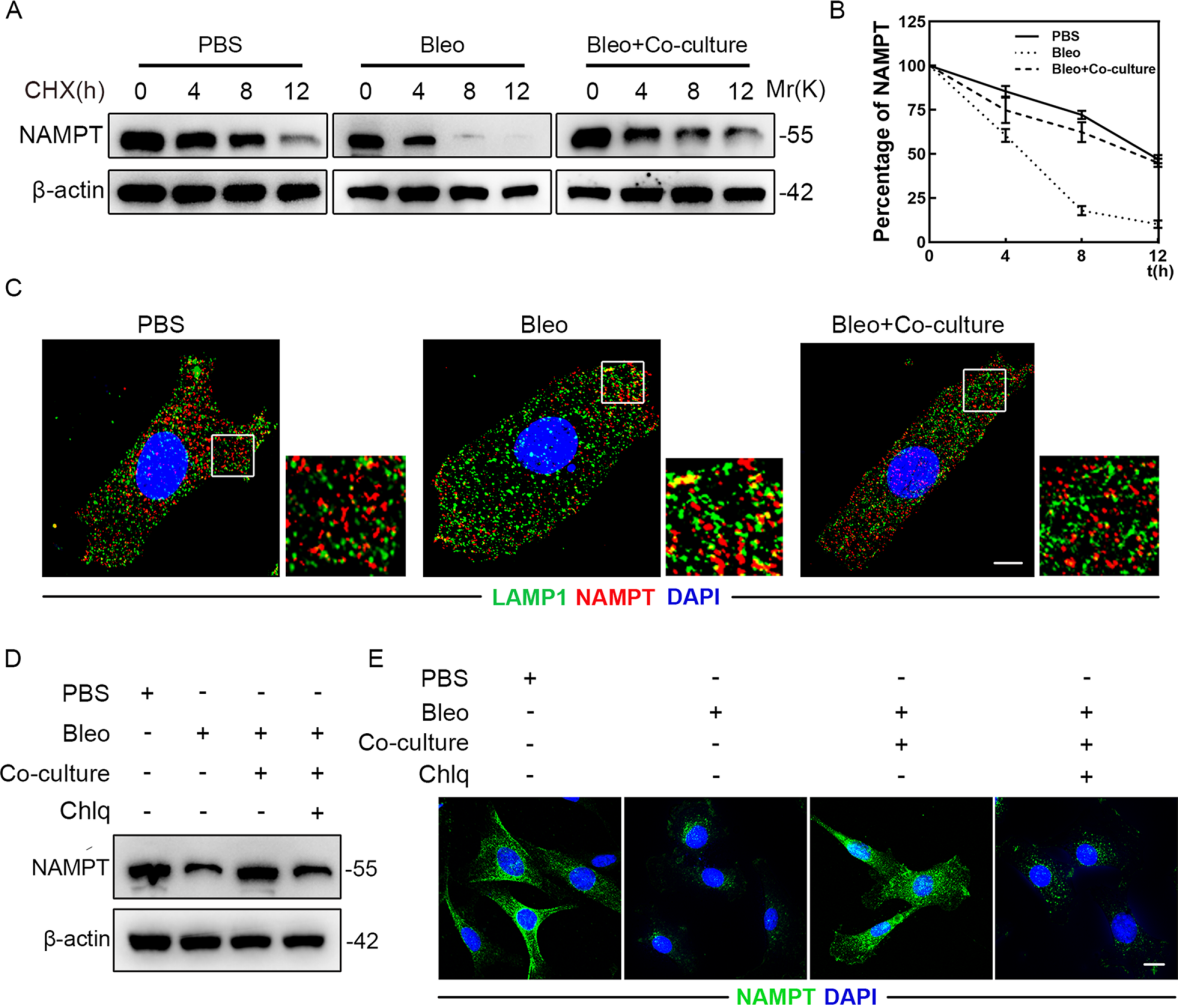
MSCs inhibit AT2 cells senescence by suppressing lysosome-mediated NAMPT degradation. **A** Half-life analysis of NAMPT in primary AT2 cells co-cultured with MSCs. All cell groups were treated with cycloheximide (CHX, 50 μg/ml) harvested at the indicated times (0, 4, 8, 12 h after CHX treatment) and subjected to immunoblotting (*n* = 3 per group). **B** Quantification of NAMPT protein levels in **A**. **C** Immunofluorescence staining of primary AT2 cells co-cultured with MSCs and visualized using anti-LAMP1 (green) and anti-NAMPT (red) antibodies. Scale bars: 10 µm. **D** Western blot analysis of NAMPT expression in primary AT2 cells co-cultured with MSCs. **E** Immunofluorescence staining of primary AT2 cells co-cultured with MSCs and visualized using anti-NAMPT (red) antibodies. Scale bars: 10 µm. Data are presented as the mean ± SEM of three independent experiments

Given that MSCs increased the protein levels of NAMPT instead of its mRNA levels in primary AT2 cells, we next asked whether MSCs could indeed modulate NAMPT stability as well as the underlying molecular mechanisms. To verify this, we treated primary AT2 cells with the protein synthesis inhibitor cycloheximide (CHX) and detected that bleomycin resulted in a shortened half-life of NAMPT in cells, which could be largely rescued by MSCs co-culture (Fig. 5A, B). Immunofluorescence staining revealed that bleomycin significantly increased the amount of NAMPT that reached LAMP1 + vesicles, which could be restored by MSCs co-culture (Fig. 5C). Moreover, MSCs stabilized NAMPT in primary AT2s but this was blocked by the lysosome inhibitor chloroquine (Chlq) (Fig. 5D, E). The above data suggested that MSCs inhibit AT2 cells senescence possibly by suppressing lysosome-mediated NAMPT degradation.

### NAMPT is required for MSCs inhibiting AT2 cells senescence

**Fig. 6:**
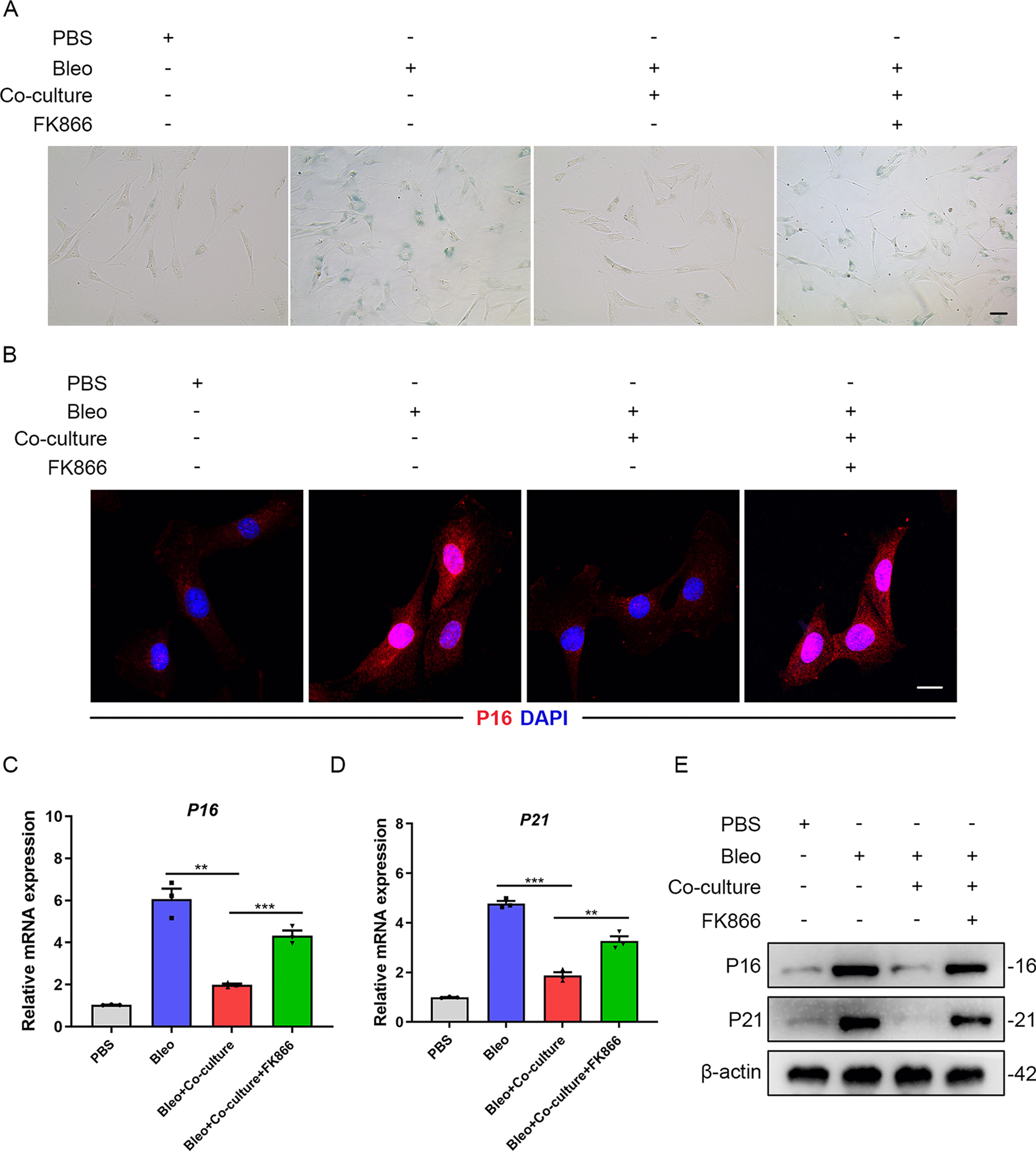
NAMPT is required for MSCs inhibiting AT2 cells senescence. **A** SA-β-galactosidase staining of primary AT2 cells co-cultured with MSCs with or without FK866 treatment. **B** Immunofluorescence staining of primary AT2 cells co-cultured with MSCs and visualized using anti-P16 (red) antibodies. Scale bars: 10 µm. **C** qPCR analysis of P16 mRNA expression in primary AT2 cells co-cultured with MSCs with or without FK866 treatment. **D** qPCR analysis of P21 mRNA expression in primary AT2 cells co-cultured with MSCs with or without FK866 treatment. **E** Western blot analysis of P16 and P21 expression in primary AT2 cells co-cultured with MSCs with or without FK866 treatment. Data are presented as the mean ± SEM of three independent experiments; **P < 0.01, ***P < 0.001; one-way ANOVA and Tukey’s multiple comparisons test

To investigate whether NAMPT plays a crucial role in MSCs inhibiting AT2 cells senescence, we tried to treat primary AT2 cells with FK866, a specific inhibitor of the NAMPT pathway [23]. SA-β-galactosidase staining showed an increase of SA-β-galactosidase positive rate in AT2 cells co-cultured with MSCs after FK866 treatment (Fig. 6A). We also found that MSCs exert an inhibitory effect on P16 expression in AT2 cells, which could be blocked by FK866 (Fig. 6B). The similar results were obtained through qPCR and Western blot analysis (Fig. 6C–E and Additional file 1: Figure S5A, B). To further confirm the results, we applied transfection with NAMPT shRNA vectors in vitro. Similarly, the results revealed that the percentage of SA-β-galactosidase positive AT2 cells co-cultured with MSCs significantly decreased, while this phenomenon did not exist after NAMPT knockdown (Additional file 1: Figure S5C). Moreover, the downregulation of P16 and P21 mRNA levels in AT2 cells co-cultured with MSCs was blocked by NAMPT knockdown (Additional file 1: Figure S5D, E). The above results further suggested that NAMPT is required for MSCs inhibiting AT2 cells senescence.

## Materials and methods

### Animal experiments

The use of animals was approved by the Ethical Committee of Sun Yat-sen University. C57BL/6 mice were purchased from Beijing Vital River Laboratory. All mice were provided with free access to food and water, and kept in a colony room under a 12-h dark–light cycle in the Sun Yat-sen University Animal Center. In our study, age- and weight-matched groups of mice were used. For establishing pulmonary fibrosis models, 8-week-old C57BL/6 mice were anesthetized with isoflurane and injected with bleomycin (Teva Pharmaceutical; 3 U/kg) or an equal volume of PBS intratracheally using a 30-gauge needle. MSCs (5 × 10^5^ cells in 50 μl PBS) were injected via the caudal vein after bleomycin administration. All animals were sacrificed 21 days after bleomycin administration.

### Cell isolation and culture

Human bone marrow aspirates were obtained from healthy donors with their informed consents. MSCs were collected from human bone marrow, cultured and identified as previously described [24]. Human MSCs exhibited surface expression of CD73, CD90 and CD105 but not CD34, CD31 or CD45. The forced differentiation into osteoblasts, chondrocytes or adipocytes was adopted to confirm the multipotent differentiation capacity of MSCs (Additional file 1: Figure S1A, B). All healthy donors signed informed consent for their samples to be used for research, and approval was obtained from the Committees for Ethical Review of Research.

Mouse lung tissue was digested with collagenase type I (#17018029, ThermoFisher) (Diluted with HBSS, in supplement with 0.1% BSA) and then filtered through a 40 µm sieve successively. Dissociated single-cell preparations were stained using corresponding antibodies listed in Additional file 1: Table S1. BD Influx cell sorter was used for fluorescence-activated cell sorting (FACS) and AT2 cells were identified as CD31-/CD45-/EpCAM + /Sca-1 + . Human A549 cells were purchased from American Type Culture Collection (ATCC). Cycloheximide (CHX) and Chloroquine (Chlq) were purchased from Sigma-Aldrich.

### Lentiviral vector transduction

pLV/puro-EF1a-tdTomato was used to transduce injected MSCs. 293FT cells were cotransfected with Lipofectamine 2000 (Invitrogen) and obtained lentivirus particles were filtered through a 0.45-μm cell strainer. MSCs were transduced with the harvested lentivirus and purified through a flow cytometer (Influx, Becton Dickinson).

### Histopathological evaluation

After sacrifice, mouse lung tissues were collected, fixed with 4% paraformaldehyde and then subjected to paraffin embedding or saturated with 30% sucrose for 24 h for frozen sections. Paraffin-embedded lung tissue sections were exposed to H&E, Masson trichrome and Sirius red staining to analyze inflammation and fibrosis in the lung tissues.

### Immunofluorescence staining (IF)

Following deparaffinization and antigen retrieval, the tissue specimens were incubated overnight at 4◦C with corresponding primary antibodies. Nuclei were visualized with DAPI (Fluka). Images were obtained using a Zeiss 800 Laser Scanning Confocal Microscope and a Zeiss 880 Laser Scanning Confocal Microscope with Airyscan. The utilized primary and secondary antibodies are listed in Additional file 1: Table S1.

### Hydroxyproline assay

Mouse lung tissue samples from mice were incubated in 250 μl PBS and then 250 μl of 12 N HCl was added into samples at 110 °C overnight. 10 N NaOH was added into the samples. 100 μl of each sample was mixed with 400 μl oxidizing solution (1.4% chloramine-T, 10% N-propanol, and 80% citrate-acetate buffer in PBS) and then incubated for 20 min. After adding Ehrlich’s solution (Sigma-Aldrich), samples were finally incubated for 30 min at 65 °C. The reaction absorbance was measured at 550 nm. The sample concentration was determined according to the standard curve generated with trans-4-hydroxy-l-proline (sigma-Aldrich).

### Real-time quantitative PCR

TRIzol reagent (Molecular Research Center, Inc.) was used to extract the total RNA from tissues or cells using the TRIzol reagent according to the manufacturer's protocol. Corresponding quantification was performed with a NanoDrop 8000 spectrophotometer. One µg RNA was used for reverse transcription with a RevertAid First Strand cDNA Synthesis Kit (Thermo Fisher Scientific, K1622). The real-time quantitative PCR (qPCR) reactions were conducted using the obtained cDNAs as the template with the FastStart Essential DNA Green Master Mix (Roche, 06924204001). The results were normalized with respect to that of 18S rRNA or GAPDH. The primers used for qPCR are listed in Additional file 1: Table S2.

### Western blotting

Collagen I, α-SMA, P16, P21, NAMPT and β-actin protein expression in lung tissue samples and cells were measured by Western blotting as previously described [25]. All the antibodies used are listed in Additional file 1: Table S1. All the Western blotting experiments were performed at least three times and the bands were quantified using Image J software.

### NAD+ quantitation

Quantification of NAD+ was conducted with SIGMA NAD/NADH Quantitation kit (MAK037) following the manufacturer instructions. Briefly, 1 × 10^5^ cells were harvested for total NAD extraction. NAD+ and NADH were recognized by the NAD Cycling Enzyme Mix. The absorbance at 450 nm (A450) was measured. The NAD+ concentration was obtained by subtracting NADH from NADtotal.

### Senescence-associated β-galactosidase staining

β-galactosidase staining was conducted following the instructions from the senescence-associated β-galactosidase staining kit (Beyotime Biotechnology). Briefly, the cells were fixed with SA-β-gal staining stationary solution at room temperature for 15 min. After washing for three times, the cells were added with the pre-prepared dyeing working liquid and incubated overnight without CO2 at 37 °C. The cells were observed and imaged under a microscope (Leica DMi8) on the next day.

### Statistics

Results are expressed as the mean ± SEM of at least three independent experiments. Statistical analysis between two groups was performed by unpaired *t* test. Statistical analysis between multiple groups was performed by one-way ANOVA, with Tukey’s multiple comparison test. *P* < 0.05 was considered statistically significant, with significance defined as *P* < 0.05 (*), *P* < 0.01 (**), *P* < 0.001 (***).

## Discussion

Idiopathic pulmonary fibrosis (IPF) is a progressive and chronic interstitial lung disease with high prevalence and mortality [26], and its management remains an ongoing challenge due to its complex pathogenic mechanisms [5] and multifactorial biological processes. Abundant studies on experimental pulmonary fibrosis demonstrated that MSCs administration exerts a protective role in lung injury and fibrosis, which indicated the therapeutic potential of MSCs for treating IPF [12]. However, the mechanism underlying the MSCs-mediated alleviation of pulmonary fibrosis is still incompletely clear especially the role of MSCs in AT2 cells senescence. In our study, we focused on the mechanistic processes underlying the ability of MSCs to reverse AT2 cells senescence and bleomycin-induced pulmonary fibrosis. We provide new evidence that MSCs could inhibit AT2 cells senescence by suppressing lysosome-mediated NAMPT protein degradation.

Now MSCs-based cell therapy is a potential therapeutic strategy for treating multiple lung diseases [27]. Nicholas R Forsyth et.al reported that MSCs have a strong migratory response to alveolar epithelial cell injury and secrete several proteins including fibronectin, lumican and periostin, thus promoting alveolar epithelial cells wound repair [28]. Hua Shao et.al proved that transplantation of adipose-derived MSCs attenuates silicosis-induced pulmonary fibrosis via regulating inflammatory and apoptotic processes in rats [14]. It is well recognized that administration of hypoxia-preconditioned MSCs could attenuate bleomycin-induced pulmonary fibrosis and these effects were mainly due to MSCs-derived HGF. MSCs suppressed BiP expression and attenuated ER stress through modulating the PERK-Nrf2 signaling pathway in murine bleomycin model [15]. Recently, Noboru Hattori’s group found that serum-free media MSCs significantly suppressed bleomycin-induced pulmonary inflammation and fibrosis through increasing the number of regulatory T cells in the lungs [29]. Here we demonstrate that MSCs could apparently inhibit AT2 cells senescence in vitro and in vivo. After treated with FK866, bleomycin-induced AT2 cells senescence could not be improved by MSCs co-culture, which suggested that NAMPT is required for MSCs inhibiting AT2 cells senescence. Our findings delineate the novel mechanisms of MSCs protection in animal models of chronic interstitial lung disease.

Accumulating evidence implicates the important role of NAD+ balance in aging and age-associated diseases [30–32]. It was reported that intracellular NAD+ concentrations decrease during aging and senescence in animals [31, 32]. NAMPT, the rate-limiting enzyme in the NAD+ salvage pathway, has been proven to contribute to the increase of tissue NAD+ levels [33]. Mice with a high-fat diet showed a reduced expression of NAMPT as well as reduced activity of the NAD+ salvage pathway, which demonstrates that NAMPT expression can be inhibited during inflammation [31]. Whereas another study reported that NAMPT was significantly upregulated in response to lipopolysaccharide-induced inflammation [34]. Decreased NAMPT expression were also observed in several organs such as liver [35], adipose tissue [36], and skeletal muscle [37] during aging. NAMPT showed a broad spectrum of effects in a number of diseases including metabolic disorders, inflammatory diseases, aging and so on [38]. In the present study, we found that NAD+ and NAMPT levels are reduced in bleomycin-treated AT2 cells, which could be restored when co-cultured with MSCs in vitro. Mechanistic studies revealed that MSCs protect against the senescence of AT2 cells by suppressing lysosome-mediated NAMPT degradation. Given the complex role of NAMPT in pulmonary disorders, whether NAMPT is involved in some pathophysiologic processes in other cell populations needs to be clarified in future studies.

There were also several limitations in the present study. First, we found that MSCs co-culture could upregulate the NAMPT and NAD+ levels in AT2 cells, thus exerting an inhibitory effect on AT2s senescence. However, whether MSCs influence NAD metabolism and NAMPT protein levels in other cell types should be further explored in the future. In addition, we have determined that the positive rate of senescent AT2 cells co-cultured with MSCs significantly decreased, while this phenomenon did not exist after NAMPT knockdown by NAMPT gene regulation technology and a specific inhibitor in vitro. However, conditional knockout animal model might be better to further confirm the specific role of NAMPT in MSCs therapeutic effects in vivo. Moreover, we proved that MSCs suppress AT2 cells senescence by inhibiting lysosome-mediated NAMPT degradation. It will be interesting to investigate the further molecular mechanisms about how MSCs regulate the protein degradation of NAMPT through lysosomes in future studies.

In conclusion, we demonstrated that MSCs attenuate experimental pulmonary fibrosis through reducing AT2 cells senescence. Our findings highlight the importance of the NAMPT-mediated NAD+ homeostasis which may be responsible for the ability of MSCs to control chronic interstitial lung disorders.

## Supplementary Information

**Additional file 1**. Supporting information.

## Data Availability

The authors agree to share data and materials related to this manuscript.

## Abbreviation

IPF: Idiopathic pulmonary fibrosis
MSCs: Mesenchymal stromal cells
AT2 cells: Alveolar type 2 cells
IL: Interleukin
SASP: Senescence-associated secretory phenotype
PBS: Phosphate-buffered saline
H&E: Hematoxylin and eosin
NAD: Nicotinamide adenine dinucleotide
