# Correction: Mesenchymal stromal cells attenuate alveolar type 2 cells senescence through regulating NAMPT‑mediated NAD metabolism

**DOI:** 10.1186/s13287-022-02869-1

**Published:** 2022-04-29

**Authors:** Xiaofan Lai, Shaojie Huang, Sijia Lin, Lvya Pu, Yaqing Wang, Yingying Lin, Wenqi Huang, Zhongxing Wang

**Affiliations:** 1 Department of Anesthesiology, The First Affiliated Hospital, Sun Yat-sen University, Guangzhou, China; 2 Zhongshan School of Medicine, Sun Yat-sen University, Guangzhou, China

## Correction to: Stem Cell Research & Therapy (2022) 13:12 10.1186/s13287-021-02688-w

Following the publication of the original article [1], the authors identified an error in Fig. 2. The authors noticed that the image of Bleo + MSCs group was not the representative image in Fig. 2A, which was accidentally used during the layout of figures. It has been corrected after they double checked the original data. The results and conclusion concluded in this paper are still valid.

**Fig. 2:**
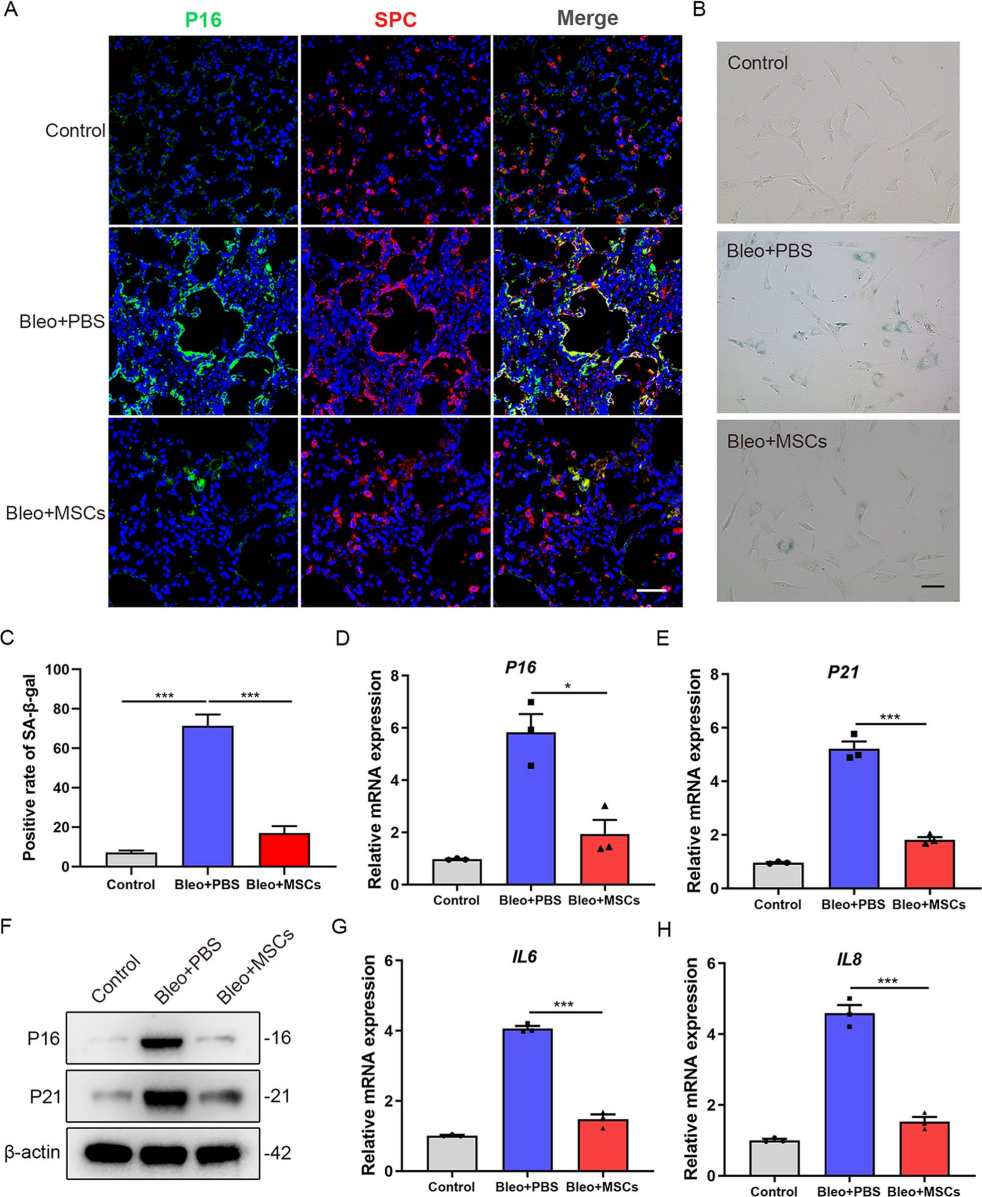
Senescence markers are downregulated in AT2 cells of MSCs-treated pulmonary fibrosis mice. **A** Immunofluorescence staining of lung sections from mice (*n* = 6 per group) and visualized using anti-P16 (green) and anti-SPC (red) antibodies. Scale bars: 50 μm. **B** SA-β-galactosidase staining of primary AT2 cells from mice of the different groups (*n* = 6 mice per group). **C** Quantification of the percentage of β-galactosidase positive cells from B. **D** qPCR analysis of P16 mRNA expression in primary AT2 cells from mice of the different groups. **E** qPCR analysis of P21 mRNA expression in primary AT2 cells from mice of the different groups. **F** Western blot analysis of P16 and P21 expression in primary AT2 cells from mice of the different groups. **G** qPCR analysis of IL6 mRNA expression in primary AT2 cells from mice of the different groups. **H** qPCR analysis of IL8 mRNA expression in primary AT2 cells from mice of the different groups. Data are presented as the mean ± SEM of three independent experiments; *P < 0.05, ***P < 0.001; one-way ANOVA and Tukey’s multiple comparisons test
