# Supplementary material for: Mesenchymal stromal cells attenuate alveolar type 2 cells senescence through regulating NAMPT-mediated NAD metabolism
Source: Stem Cell Res Ther. 2022 Jan 10;13:12. doi: 10.1186/s13287-021-02688-w (PMC8751376; doi:10.1186/s13287-021-02688-w)
Supplement: Supplementary file 1 — Additional file 1. Supporting information. [file 13287_2021_2688_MOESM1_ESM.docx]

**Mesenchymal Stromal Cells Attenuate Alveolar Type 2 Cells Senescence Through Regulating NAMPT-mediated NAD Metabolism**

Xiaofan Lai, Shaojie Huang, Sijia Lin, Lvya Pu, Yaqing Wang, Yingying Lin, Wenqi Huang, Zhongxing Wang


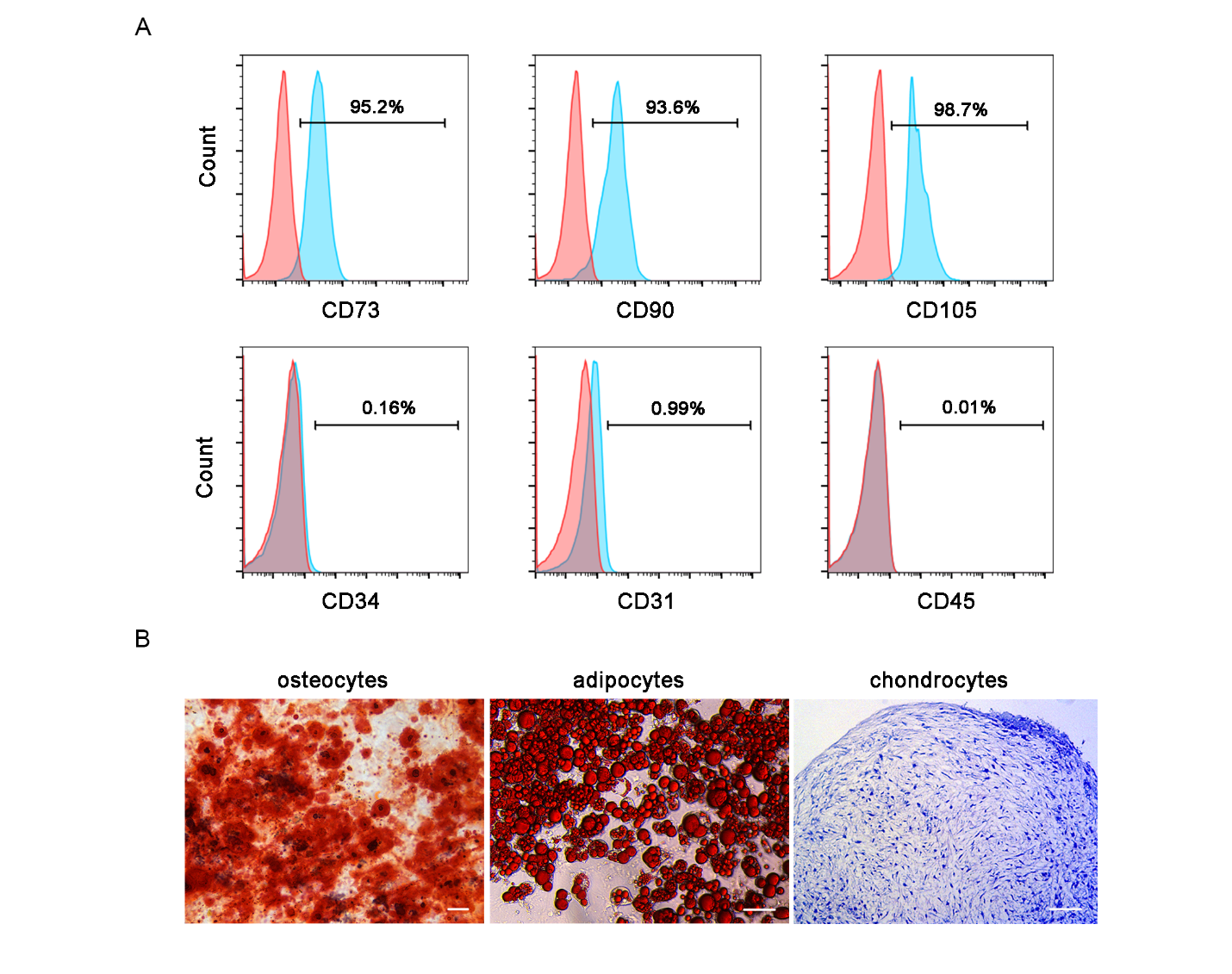


Figure S1. The characteristics of human MSCs in vitro.

**(A)** The MSCs surface expression levels of CD73, CD90, CD105, CD34, CD31 and CD45 analyzed by flow cytometry. **(B)** Alizarin red S, Oil red O and Toluidine blue O staining were used to assess the adipogenic, osteogenic, and chondrogenic differentiations of MSCs respectively. Scale bar=100 um.


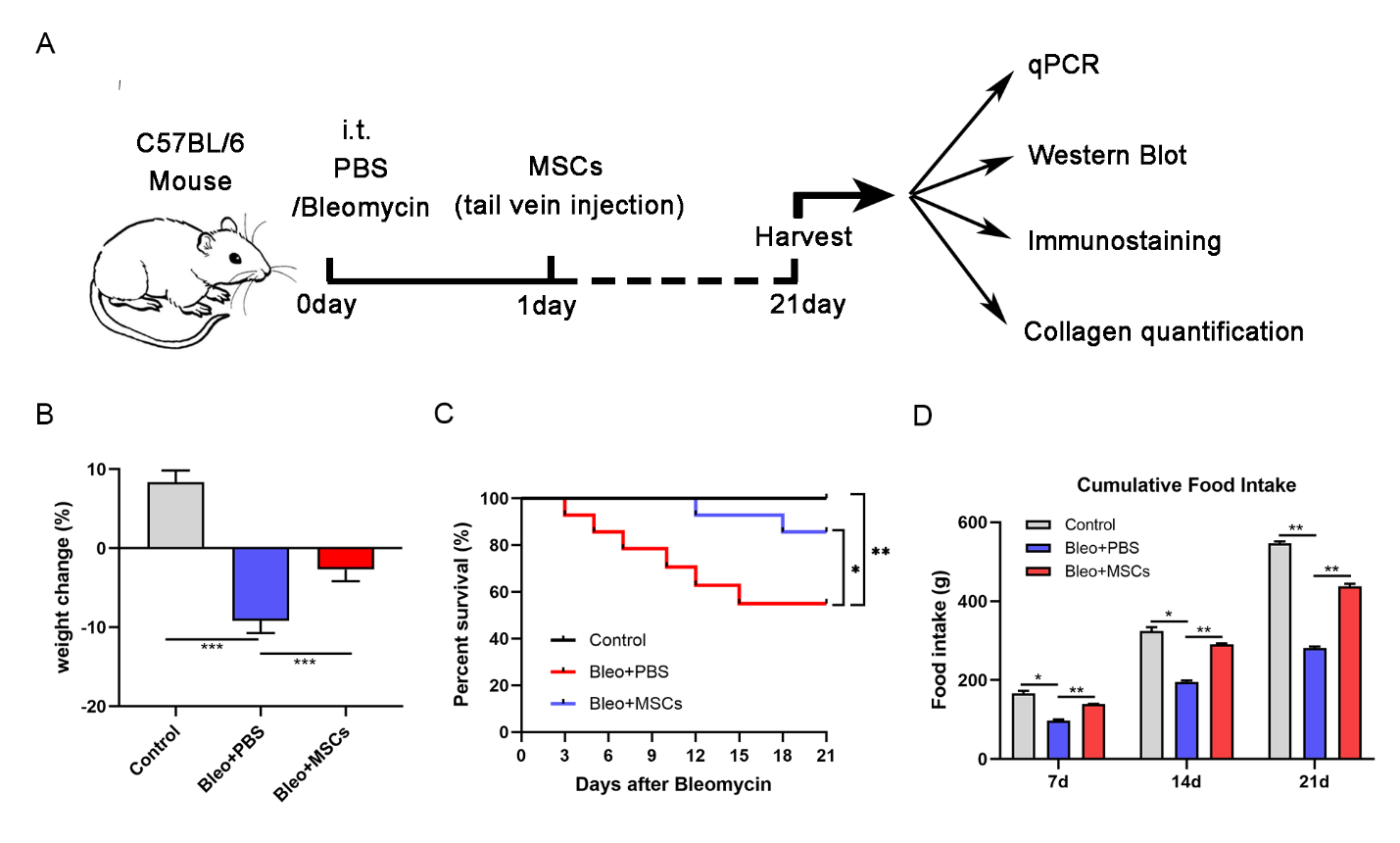


Figure S2. MSCs attenuate bleomycin-induced pulmonary fibrosis in mice.

**(A)** Experimental design. 8-week-old C57BL/6 mice were intratracheally injected with bleomycin (3U/kg) or PBS. MSCs (5 × 10^5^ cells in 50 μl PBS) were injected via the caudal vein one day after bleomycin administration. Samples were collected for analysis 21 days after bleomycin administration. **(B)** Body weight change (%) are presented at 21 days after bleomycin injection. **(C)** Survival rates (percent survival) are presented at 3-day intervals (n = 14 mice per group). Data are presented as the mean ± SEM; *P<0.05, **P<0.01, ***P<0.001; one-way ANOVA and Tukey’s multiple comparisons test.


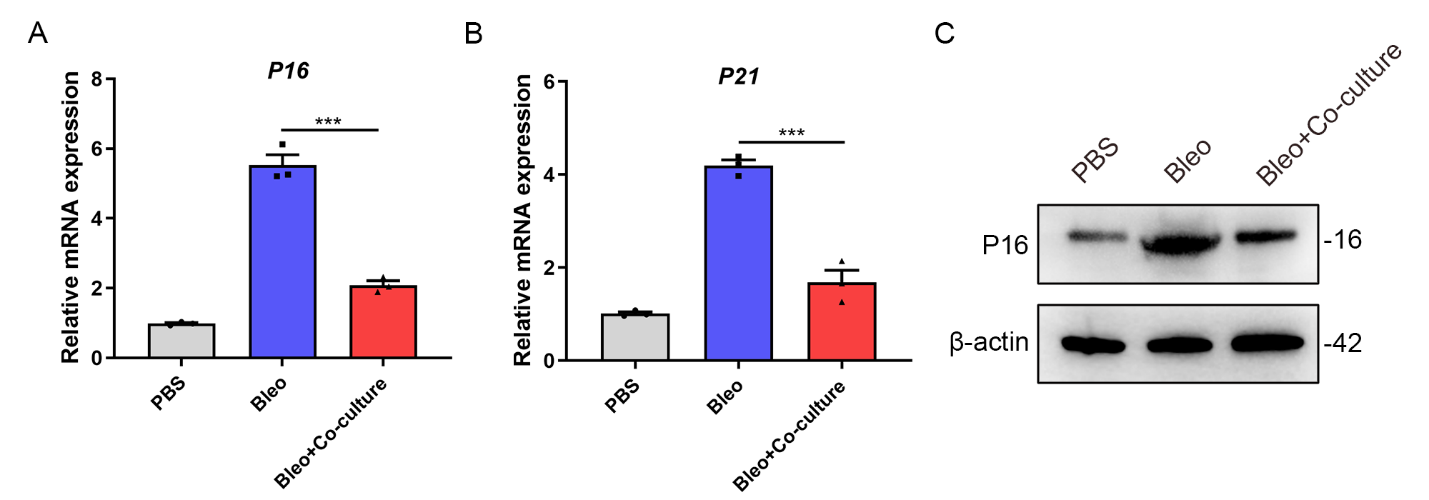


**Figure S3** A549 cells co-cultured with MSCs decreases senescent markers.

**(A)** qPCR analysis of P16 mRNA expression in A549 cells co-cultured with MSCs. **(B)** qPCR analysis of P16 mRNA expression in A549 cells co-cultured with MSCs. **(C)** Western blot analysis of P16 expression in A549 cells co-cultured with MSCs. Data are presented as the mean ± SEM; ***P<0.001; one-way ANOVA and Tukey’s multiple comparisons test.


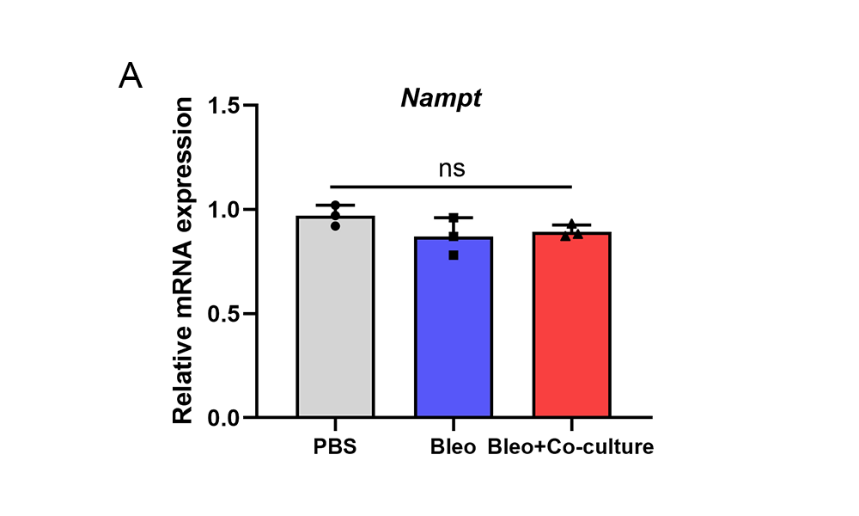


**Figure S4** NAD+ and NAMPT levels are restored in primary AT2 cells co-cultured with MSCs. **(A)** qPCR analysis of Nampt mRNA expression in primary AT2 cells co-cultured with MSCs. Data are presented as the mean ± SEM; ns: no significance; one-way ANOVA and Tukey’s multiple comparisons test.


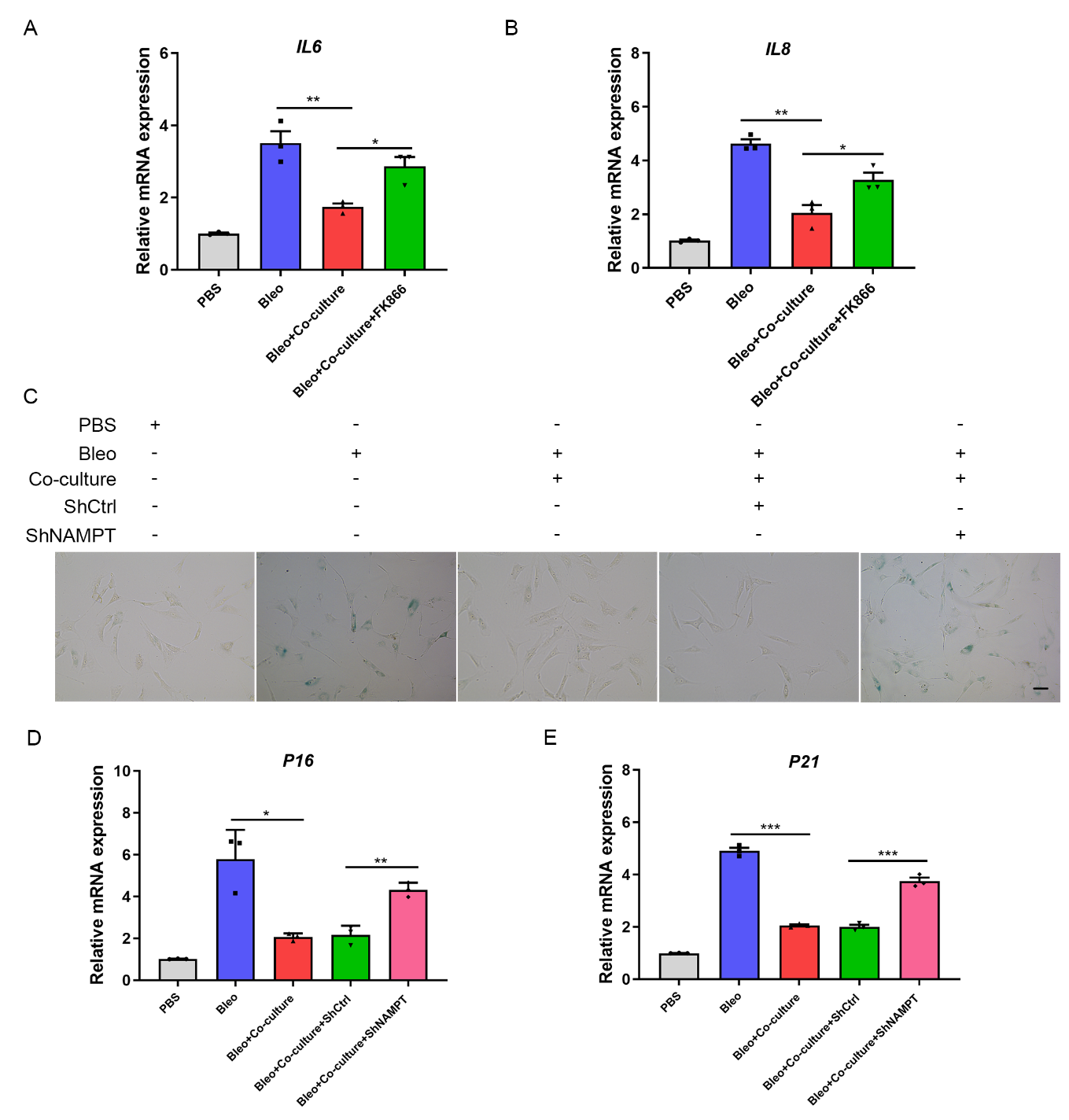


**Figure S5** NAMPT is required for MSCs inhibiting AT2 cells senescence.

**(A)** qPCR analysis of IL6 mRNA expression in primary AT2 cells co-cultured with MSCs with or without FK866 treatment. **(B)** qPCR analysis of IL8 mRNA expression in primary AT2 cells co-cultured with MSCs with or without FK866 treatment. **(C)** SA-β-galactosidase staining of primary AT2 cells co-cultured with MSCs with or without NAMPT knockdown. **(D)** qPCR analysis of P16 mRNA expression in primary AT2 cells co-cultured with MSCs with or without NAMPT knockdown. **(E)** qPCR analysis of P21 mRNA expression in primary AT2 cells co-cultured with MSCs with or without NAMPT knockdown. Data are presented as the mean ± SEM; *P<0.05, **P<0.01, ***P<0.001; one-way ANOVA and Tukey’s multiple comparisons test.

**Table S1. Primary and secondary antibodies.**

| **Product** | **Catalog Number** | **Supplier** |
| --- | --- | --- |
| **Primary antibody**: |  |  |
| rabbit anti-NAMPT | 11776-1-AP | Proteintech |
| mouse anti-α-SMA | ab7817 | Abcam |
| rabbit anti-Collagen I  mouse anti-β-actin  rabbit anti-Prosurfactant Protein C  rat Anti-LAMP1  rabbit Anti-P16  rabbit Anti-P21  rabbit Anti-P21  **Flow:**  PE-Cyanine7 Rat Anti-Mouse CD31  PE-CF594 Rat Anti-Mouse CD45  Anti-Mouse CD326 (EpCAM) APC  Anti-Mouse Ly-6A/E (Sca-1) FITC  CD73 (PE)  CD90 (APC)  CD105 (PE)  CD34 (FITC)  CD45 (Pe-cy7)  CD31 (FITC) | ab260043  600081  AB3786  MABC39  ab51243  ab188224  2947S  25-0311-82  562420  17-5791-82  11-5981-85  AD2  5E10  MHCD10505  4H11  HI30  WM59 | Abcam  Proteintech  Millipore  Millipore  Abcam  Abcam  Cell Signaling Technology  eBioscience  BD Biosciences  eBioscience  eBioscience  BD Biosciences  BD Biosciences  Thermo Fisher  Thermo Fisher  Thermo Fisher  BD Biosciences |
| **Secondary antibody**:  anti-mouse IgG HRP-linked Ab  anti-rabbit IgG HRP-linked Ab  goat anti-mouse IgG Alexa 488  goat anti-rabbit IgG Alexa 488  goat anti-rabbit IgG Alexa 555  goat anti-mouse IgG Alexa 555  goat anti-rat IgG Alexa 488 | 7076  7074  A11001  A11008  A21428  A21422  A11006 | Cell Signaling Technology  Cell Signaling Technology  Invitrogen  Invitrogen  Invitrogen  Invitrogen  Invitrogen |

**Table S2.** **Primer used for q-PCR.**

| **Gene** | **Sequence (5′ to 3′)** | **Application** |
| --- | --- | --- |
| m*P16*  m*Nampt* | Forward: 5’-CGGGGACATCAAGACATCGT-3’  Reverse: 5’-GCCGGATTTAGCTCTGCTCT-3’  Forward: 5’-TGCCGTGAAAAGAAGACAGA-3’  Reverse: 5’-ACTTCTTTGGCCTCCTGGAT-3’ | qPCR  qPCR |
| m*Acta2* | Forward: 5’-TGAGACCTTCAATGTCCCCGC-3’  Reverse: 5’-TCACACCATCTCCAGAGTCCAGC-3’ | qPCR |
| m*Col1a1* | Forward: 5’-TGACTGGAAGAGCGGAGAGT-3’  Reverse: 5’-GTTCGGGCTGATGTACCAGT-3’ | qPCR |
| m*P21*  m*IL6*  m*IL8* | Forward: 5’-ACATCTCAGGGCCGAAAACG-3’  Reverse: 5’-AAGACACACAGAGTGAGGGC-3’  Forward: 5’-CCAGTTGCCTTCTTGGGACTGATG-3’  Reverse: 5’-CCGGACTTGTGAAGTAGGGAAGGC-3’  Forward: 5’-GCAACAGAAAGGAAGTGATAGCAG-3’  Reverse: 5’-AGCTTCATTGCCGGTGGAAA-3’ | qPCR  qPCR  qPCR |
| m*Fn1* | Forward: 5′-GGTGTAGCACAACTTCCAATTACG-3′  Reverse: 5′-GGAATTTCCGCCTCGAGTCT-3′ | qPCR |
| m*18S*  h*GAPDH*  h*P16*  h*P21* | Forward: 5’-GTAACCCGTTGAACCCCATT-3’  Reverse: 5’-CCATCCAATCGGTAGTAGCG-3’  Forward: 5’-GTCGGAGTCAACGGATTT-3’  Reverse: 5’-GGAATCATATTGGAACATGTAAACC-3’  Forward: 5’-ACCAGAGGCAGTAACCATGC-3’  Reverse: 5’-CCTGTAGGACCTTCGGTGAC-3’  Forward: 5’-GTCAGTTCCTTGTGGAGCCG-3’  Reverse: 5’-TGGGTTCTGACGGACATCCC-3’ | qPCR  qPCR  qPCR  qPCR |
